# An Innovative Solution to Complex Inguinal Defect: Deepithelialized SIEA Flap With Mini Abdominoplasty

**Published:** 2017-01-25

**Authors:** Augustine Reid Wilson, Justin Daggett, Michael Harrington, Deniz Dayicioglu

**Keywords:** SIEA, flap, abdominal reconstruction, inguinal reconstruction, groin

## Abstract

**Introduction:** We describe a novel technique of contralateral pedicled deepithelialized superficial inferior epigastric artery flaps, followed by abdominal advancement coverage, as an alternative treatment of radiated complicated inguinal or lower abdominal defects, avoiding the donor-site defect typically seen with other methods of coverage. **Methods:** Two male patients with histories of liposarcoma after excision and radiation to one side of lower abdomen/inguinal area presented with complicated wounds that were reconstructed with this technique. **Results:** Successful obliteration of dead space and wound closure were achieved with the combination of a superficial inferior epigastric artery flap with an abdominal advancement flap. In each case, patients went on to heal uneventfully without need for any secondary procedures. **Discussion:** The use of a superficial inferior epigastric artery flap for lower abdomen/groin defect closure is an option as an alternative to rectus abdominis myocutaneous flap and anterolateral thigh flaps and should be considered in patients with vascular anatomy conducive for this muscle-sparing procedure. **Conclusions:** A second layer-overlay coverage with an abdominal advancement flap creates a more durable repair in the complicated radiated wound and a well-concealed abdominoplasty scar.

Coverage of lower abdominal and inguinal defects can typically be achieved using advancement of excess abdominal tissues. However, in situations where a significant dead space exists or when vital structures are exposed, this may be insufficient to completely obliterate the wound. In this situation, reconstruction with pedicled flaps has been a mainstay of treatment of wounds that are not amenable to primary closure.^1^ Traditional coverage options have included locoregional flaps such as anterolateral thigh (ALT) flap and rectus abdominis muscle or myocutaneous flaps.^2^^-^5 However, each of these flap carries with it a donor-site defect in the form of potential abdominal weakness or hernia in the case of the rectus abdominis flap and visible scarring/need for a skin graft in the case of an ALT flap. We describe a novel application of a pedicled contralateral superficial inferior epigastric artery (SIEA) flap combined with an abdominal advancement flap for closure of these defects.

The SIEA/superficial inferior epigastric vein (SIEV) flap has been previously described as an option for abdominal free tissue transfer.^6^ However, this application has been limited because of the fact that while the SIEA is typically present, it is only rarely sufficiently dominant to support sufficient flap volume for breast reconstruction. As such, the vessels are frequently divided in the process of elevating a deep inferior epigastric perforators/transverse rectus abdominis myocutaneous (DIEP/TRAM) flap or an abdominal advancement flap. However, in the cases described, the SIEA/SIEV flaps are maintained to vascularize a relatively small fasciocutaneous flap from the contralateral lower abdomen, which is then advanced across the midline to fill any contralateral inguinal dead space.

## DESCRIPTION OF TECHNIQUE

1. The inguinal defect is evaluated and an ipsilateral abdominoplasty-type incision is designed, which would allow for abdominal advancement to close the wound. In the case of a chronic wound, any areas of surrounding chronic inflammation or radiation are included in the proposed excision. Negative margins are obtained.
2. The abdominal markings are then transposed to the contralateral abdominal wall. The skin island is defined by these marks, and the medial border of the defect defines the SIEA flap to be transposed.
3. Upper incision is made and carried down to abdominal wall fascia. The inferior incision is made and carried down to the abdominal wall, taking care to identify and preserve the SIEA and SIEV during the dissection. The vessels are dissected free from their surrounding tissue for 6 to 8 cm inferiorly. This dissection or the pedicle is critical for adequate advancement of the flap. A small amount of adipose tissue can be left in the area of the vessels to help protect from undue tension; however, care must be taken to adequately release all of Scarpa's fascia in order to allow for adequate mobilization. Lighted retractors are used to further dissect the pedicle more distally.
4. The flap is deepithelialized, and vascularity and bleeding are checked on all corners. It is then completely released from its deep attachment to the abdominal fascia. At no point is the anterior rectus sheath or fascia incised. This approach typically allows for a significant degree of advancement across the midline of around 10 to 12 cm or more. The deepithelialized flap is secured to the base of the contralateral inguinal wound to cover the complicated defect. The circulation is checked again after the flap is advanced and stabilized.
5. The abdominal skin flap is then undermined to the necessary level to close without tension. This abdominal skin flap reminiscent of a mini abdominoplasty flap is then advanced to close over the deepithelialized SIEA flap.
6. After drain placement, the horizontal abdominal skin closure is completed in multiple layers. This closure creates an aesthetically pleasing abdominoplasty effect, a easily hidden low scar, and a durable double-layer coverage of the complicated wound with nonirradiated tissue.

## CASE PRESENTATION

**Figure 1:**
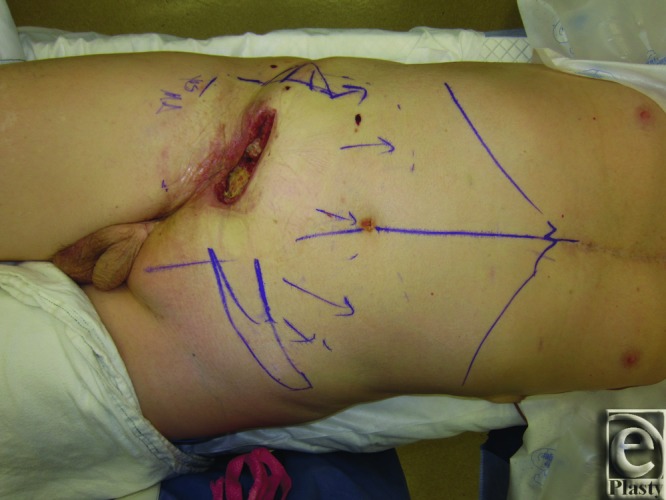
Patient 1 preoperation. The figure demonstrates the preoperative right inguinal defect and markings in patient 1.

Two patients presented after treatment of sarcomas in the lower abdomen/groin. Patient 1 was a male patient who had a 4.5-cm dedifferentiated liposarcoma resection with right lower abdominal wall/inguinal reconstruction and synthetic mesh placement, followed by radiation treatment. The patient returned with breakdown of the site, creating a draining open wound. He underwent irrigation and debridement and presented to our service for wound closure (Fig 1).

**Figure 2:**
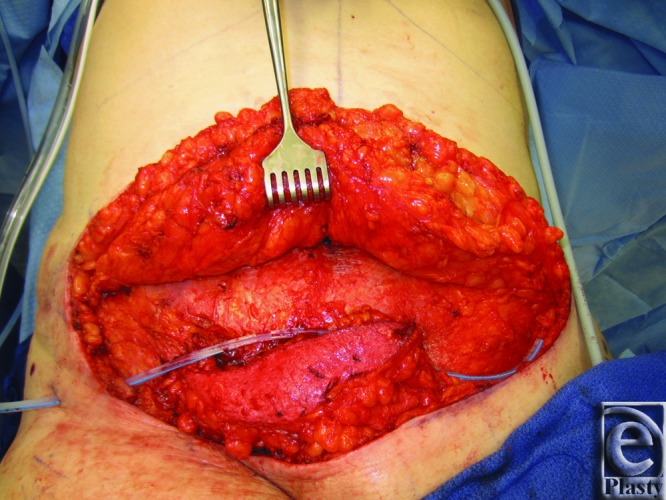
Patient 1 SIEA flap transposition. The figure demonstrates the intraoperative transposition of the left SIEA flap. SIEA indicates superficial inferior epigastric artery.

**Figure 3:**
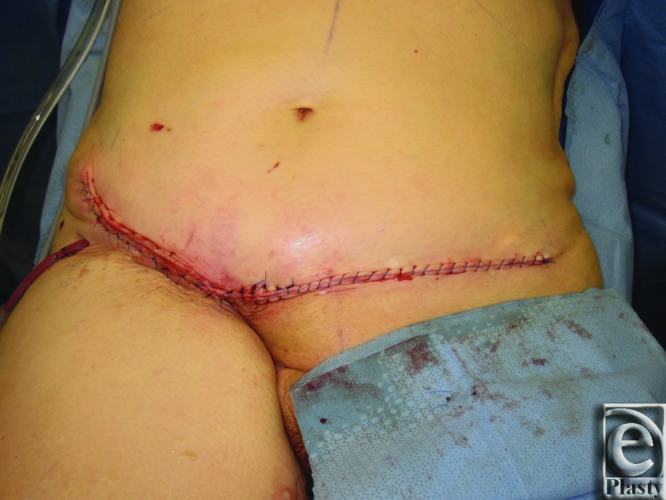
Patient 1 postoperation. The figure demonstrates the postoperative appearance in patient 1.

He underwent elevation and advancement of a left SIEA flap and closure with abdominal advancement (Figs 2 and 3). Postoperatively, he recovered uneventfully.

**Figure 4:**
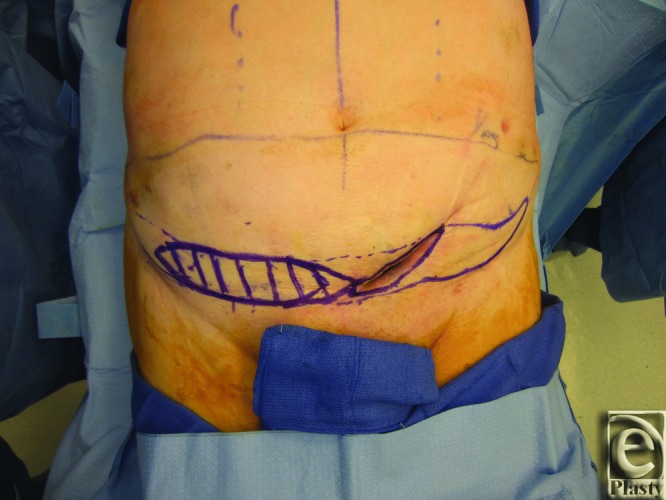
Patient 2 preoperation. The figure demonstrates the preoperative left lower abdominal defect and markings in patient 2.

Patient 2 was a male patient who underwent excision of a left lower abdominal dedifferentiated liposarcoma and rhabdomyosarcoma of left groin and suprapubic ramus, followed by radiation treatment. The patient's postoperative course was complicated by persistent seroma that required incision and drainage. He developed a nonhealing wound with significant inferior dead space before presenting to Plastic Surgery for wound closure (Fig 4).

**Figure 5:**
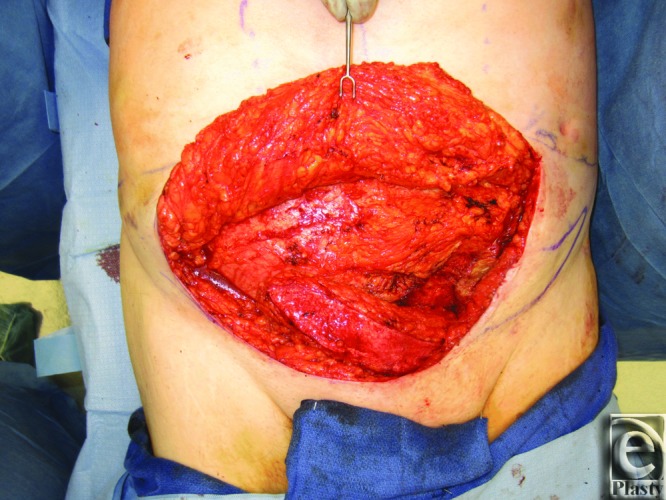
Patient 2 SIEA flap transposition. The figure demonstrates the intraoperative transposition of the right SIEA flap. SIEA indicates superficial inferior epigastric artery.

**Figure 6:**
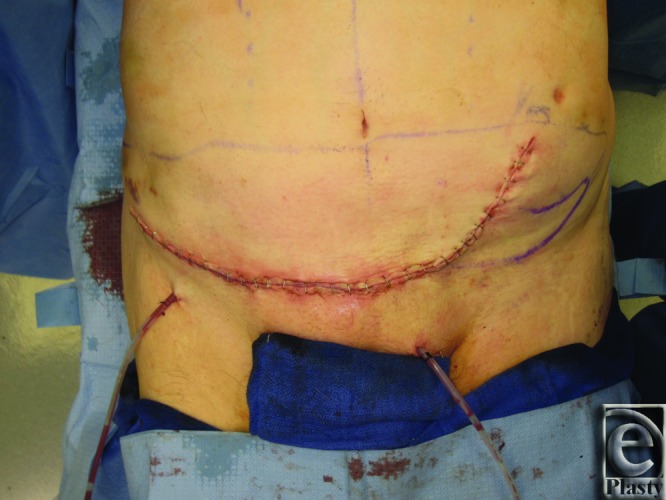
Patient 2 postoperation. The figure demonstrates the postoperative appearance in patient 2.

He underwent elevation and advancement of a right SIEA flap and closure with abdominal advancement (Figs 5 and 6). Postoperatively, he recovered uneventfully.

## DISCUSSION

The use of a SIEA flap for lower abdomen/groin defect closure provides a safe and effective alternative to rectus abdominis myocutaneous (RAM) and ALT flaps in patients with conducive vascular anatomy. Aesthetic pleasing results were obtained in these 2 cases (Figs 3 and 6) while avoiding abdominal weakness/hernia and visible scarring from skin graft seen with donor-site defects from RAM and ALT flaps, respectively. The patients described here had uneventful postoperative courses and were sitting up and out of bed working with physical therapy by postoperative day 1.

This approach eliminates a more extensive operation. It brings nonirradiated, vascularized, healthy tissue to the radiated area. While minimally invasive, it is able to cover the 3-dimensional loss of coverage of fascia and major vessels. It is technically easy; results are reproducible as long as the pedicle is present and not injured from prior surgical procedures or anatomical absence. Further closure with abdominal advancement brings a second layer of closure and creates a better scar pattern.

Limitations to this approach include viability of the contralateral SIEA/SIEV to profuse a flap of adequate size and proximity in order to fill the defect. This technique provides closure to only one side and requires viable tissue contralateral to the defect. This approach would not be viable in the presence of bilateral defects or radiation damage to the planned donor site.

In addition, in these cases, we found the pedicle allowed for advancement of about 10 to 12 cm across the midline. Larger, more lateralized defects might not be amenable to filling with this flap. Although we have had good results with this approach, it is important to note that anatomical variability of the SIEA/SIEV flap may preclude this use of the flap in some patients.^7^ Use of preoperative computed tomography angiography (CTA) would be advantageous if this was to be the planned approach for closure.

In conclusion, a deepithelialized SIEA/SIEV fasciocutaneous flap with mini abdominoplasty flap advancement is a practical, less invasive method for contralateral lower abdominal and inguinal defects.
